# Susceptibility of Yellow Clingstone Peach Cultivars to Inking Disorder

**DOI:** 10.3390/ijms27083681

**Published:** 2026-04-21

**Authors:** Paula Lopez-Raesfeld, Ma. Estela Vazquez-Barrios, Javier Añorve-Morga, Angel R. Flores-Sosa, Edmundo M. Mercado-Silva

**Affiliations:** 1 Department of Research and Graduate Studies in Food Science, Faculty of Chemistry, Autonomous University of Querétaro, Centro Universitario S/N, Cerro de las Campanas, Santiago de Querétaro 76010, Querétaro, Mexico; paula.raesfeld@gmail.com (P.L.-R.); mevazquez@uaq.edu.mx (M.E.V.-B.); 2 Faculty of Chemistry, Autonomous University of the State of Hidalgo, Ciudad del Conocimiento S/N, Carretera Pachuca–Tulancingo Km 4.5, Carboneras, Pachuca de Soto 42184, Hidalgo, Mexico; anorvej@uaeh.edu.mx; 3 Faculty of Chemistry, Pedro Escobedo Campus, Autonomous University of Querétaro, Panamericana Avenue No. 180 Int. 100, Centro, Pedro Escobedo 76700, Querétaro, Mexico; angel.ramon.flores@uaq.mx

**Keywords:** inking disorder, mechanical damage, metal ions, peach fruit, cultivar dependence

## Abstract

Inking disorder is a peach quality defect in which fruits manifest a dark epidermis discoloration; this disorder reduces the commercial value of peach fruits. In red pigmented peach fruit, it has been proposed that inking is associated with mechanical damage and reactions involving metal ions (Fe, Cu, and Zn) and anthocyanins, but in yellow peach cultivars inking mechanism is unknown. The objective of the present investigation was to evaluate the effect of mechanical damage and contamination with Fe metal ions in the development of inking disorder in three yellow peach cultivars. The present study revealed that Fe_2_(SO_4_)_3_ application increased total phenolic content and chlorogenic acid levels in all cultivars but did not induce inking symptoms in the absence of mechanical damage. In contrast, brushing treatments triggered inking development in ‘Colegio’ and ‘MG8’, allowing differentiation of cultivar susceptibility, whereas ‘229’ showed complete resistance. Mechanical damage also significantly increased phenolic compounds, as well as phenylalanine ammonia-lyase (PAL) and polyphenol oxidase (PPO) activities, with higher levels consistently observed in susceptible cultivars. Notably, the severity of inking increased when mechanical damage was combined with Fe(III) application. These results indicate that inking development requires both phenolic accumulation and their enzymatic oxidation. Fe(III) ions act as an enhancing factor by stimulating phenolic biosynthesis, thereby intensifying discoloration. The resistance observed in cv. ‘229’ is associated with lower phenolic synthesis and oxidative activity. This study provides new insights into the physiological mechanisms of inking in yellow peaches and offers practical implications for postharvest management and cultivar selection.

## 1. Introduction

Peach is a source of carbohydrates, potassium, phosphorus and bioactive compounds, and its world production increased 25% in the 2010–2020 decade, reaching 25,737,841 tons in 2020 [1]. More than 50% of total peach production is distributed as fresh fruit; therefore, external appearance is an important factor to ensure consumer acceptance [2,3].

One of the principal defects that negatively affects the visual quality of peach fruit is the inking disorder. This disorder is characterized by the presence of dark brown spots on the fruit epidermis, generating rejections in all marketing chains and economic losses in peach industry [4]. Inking development has been associated with contamination of metal ions such as Fe, Cu or Al in alkaline water and mechanical damage [5,6,7,8]. It has been proposed that the mechanical damage disrupts cell compartmentalization, allowing the interaction between phenolic compounds and metal ions; this interaction may form dark-colored metal–phenolic complexes, which are associated with the characteristic discoloration observed in inked fruit [5,6,8,9].

Most available information on inking has been derived from studies on red-pigmented freestone peaches and nectarines cultivars, in which anthocyanins play a central role in discoloration processes [7,9]. In contrast, yellow clingstone peaches do not synthesize anthocyanins, suggesting that alternative mechanisms, involving non-pigmented phenolic compounds, are responsible for inking development in these cultivars. Furthermore, marked cultivar-dependent differences in susceptibility to inking have been reported, indicating that intrinsic physiological traits may critically influence the disorder development [4,7]. The objective of this research was to identify the relationship and role of Fe III ions on fruit epidermis and the mechanical brushing on the inking disorder development in three yellow peach cultivars (‘MG8’, ‘Colegio’, and ‘229’).

## 2. Results and Discussion

### 2.1. Participation of Fe(III) Ions into Inking Disorder

**Figure 1:**
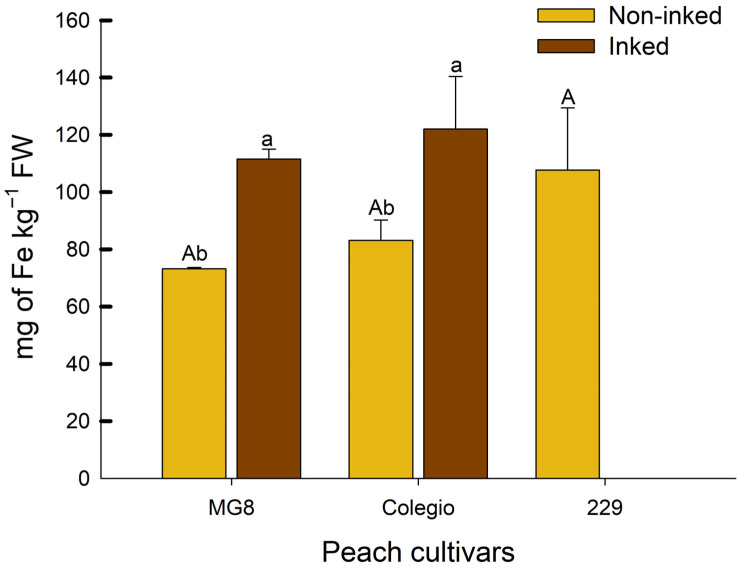
Fe content on epidermis from inked and non-inking peach fruit from different cultivars. Graphs depict mean values ± SE (*n* = 3). Capital letters indicate statistical comparison among different cultivars using one-way ANOVA and Tukey test (*p <* 0.05); lowercase letters indicate statistical comparison between epidermis of inked and non-inked within the same cultivar using *t*-test (*p <* 0.05).

Mineral analysis showed that Fe content was higher in the epidermis of inking fruits in comparison with the epidermis of non-inking peaches (Figure 1); the content of other mineral elements (Zn, Mn, Cu, B, Ni, and Mo) is presented in Supplementary Table S1. Among these, Zn, Cu, B, and Ni in one or both cultivars showed differences between inked and non-inked fruits; however, these variations were considerably lower than those observed for Fe (Table S1). In inking epidermis from ‘MG8’ and ‘Colegio’ cultivars, the Fe content increased by ~50% compared with non-inking epidermis. This suggests that increases in Fe content could participate in the inking development of yellow peach cultivars. Similar observations have been reported by other authors; Cheng and Crisosto [7] demonstrated that red pigmented peach cultivars with incidence of this disorder had higher Fe contents than fruits without the disorder. Importantly, this disorder was not observed on fruit from cv. ‘229’ during field sampling, so we could consider this cultivar to be resistant to the inking phenomenon.

**Figure 2:**
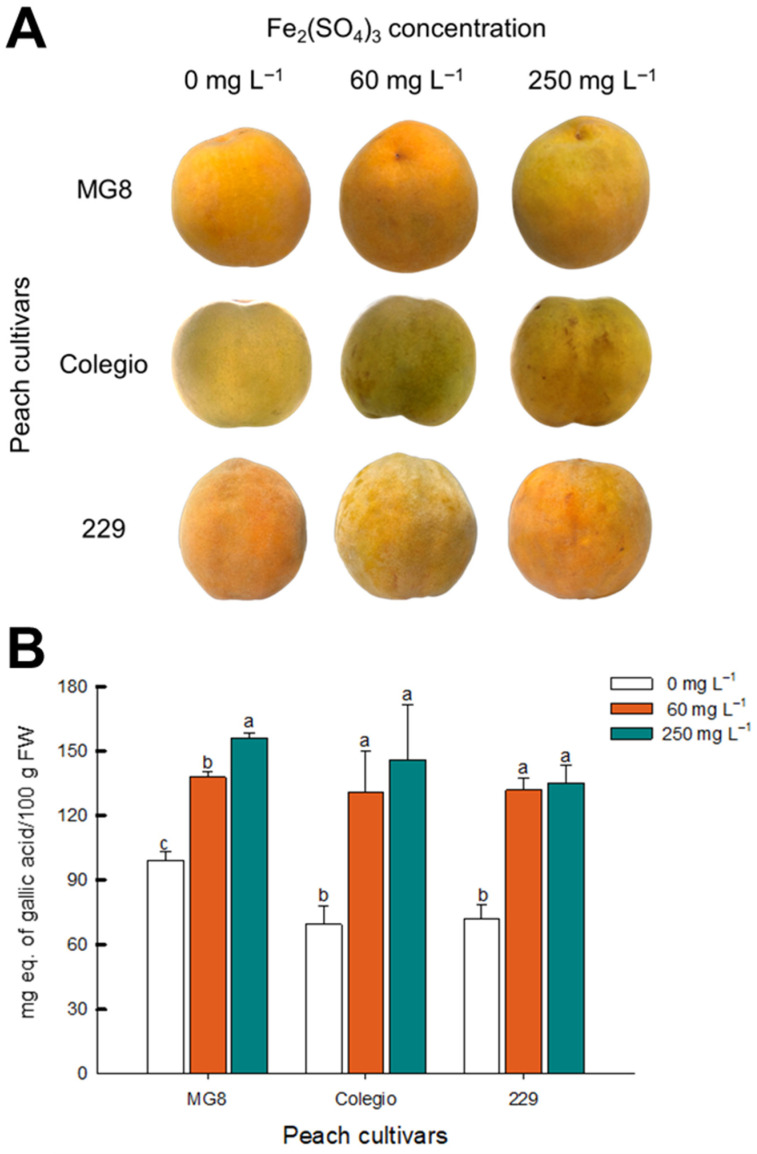
Visual appearance (**A**) and total phenols content (**B**) in epidermis samples from different cultivars of clingstone yellow peaches after application of 0, 60, and 250 mg L^−1^ of Fe_2_(SO_4_)_3_. Graphs depict mean values ± SE (*n* = 3). Letters indicate statistical comparison within the same cultivar using one-way ANOVA and Tukey test (*p* < 0.05).

To corroborate if the presence of Fe(III) ions on fruit epidermis induces the inking phenomenon, fruit samples were sprayed with different concentrations of Fe_2_(SO_4_)_3_ solutions (0 to 250 mg L^−1^). Visual inspection revealed that the presence of Fe(III) ions did not induce inking on the fruit epidermis (Figure 2A). This observation was supported by color analysis (Table S2); a decrease in L* values is indicative of increased surface darkening, corresponding to the development of inking symptoms. However, in the three cultivars, the application of Fe(III) solutions induced an increase in the biosynthesis of phenolic compounds (Figure 2B). These results indicate that Fe contamination by itself does not generate inking appearance but induces the accumulation or biosynthesis of phenolic compounds, as described elsewhere [10,11], which, together with other factors such as mechanical damage, could induce the dark spots typical of inking.

### 2.2. Mechanical Damage as a Factor to Enhance the Inking Presence

**Figure 3:**
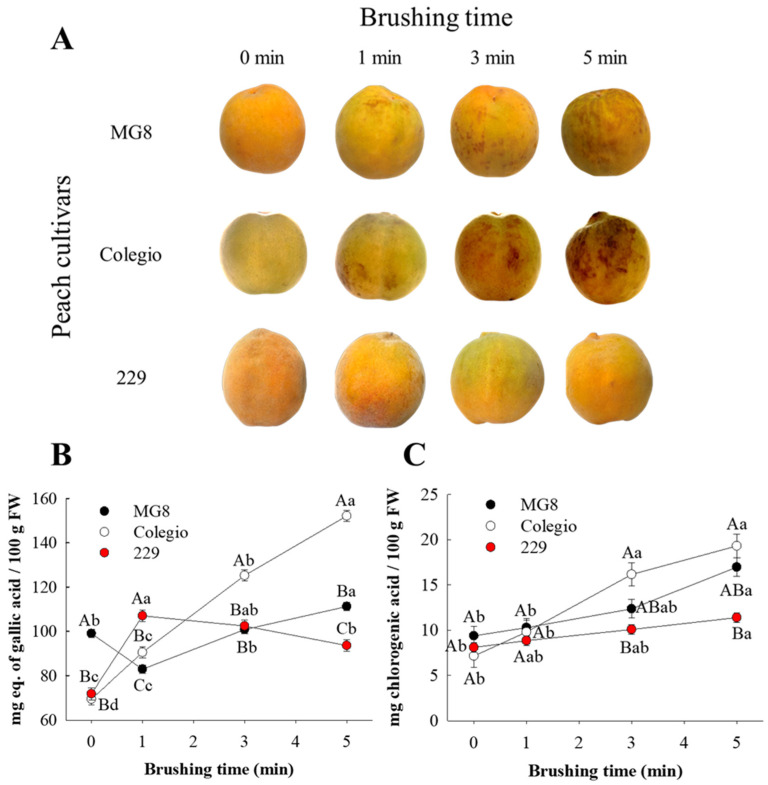
Changes in visual appearance (**A**), total phenolic content, TPC, (**B**) and chlorogenic acid (**C**) in yellow clingstone peach epidermis from different cultivars. Graphs depict mean values ± SE (*n* = 3). Letters indicate statistical comparison in the three cultivars using one-way ANOVA and Tukey test (*p <* 0.05). Different capital letters indicate statistical differences between cultivars and lowercase letters indicate statistical differences within the same cultivar.

To evaluate if mechanical damage is linked with inking disorder in yellow peach cultivars, samples were brushed for different times and then analyzed. Visual and color analysis revealed that mechanical damage during 1 min generates an inking defect in cv. ‘Colegio’, while treatments during 3 and 5 min induced an inking appearance in the fruit epidermis of ‘MG8’ and ‘Colegio’ cultivars (Figure 3A and Table S3); importantly, inking disorder was not observed in fruits from cv. ‘229’. Therefore, this analysis corroborates that mechanical damage is associated with the presence of inking disorder in ‘MG8’ and ‘Colegio’ cultivars. These findings are consistent with previous reports indicating that mechanical damage is closely associated with inking development and other visual defects in different fruit species [5,6,12]. Also, this experiment allowed us to distinguish susceptibility to inking defect among yellow peach cultivars; it was observed that ‘Colegio’ and ‘MG8’ were identified as susceptible cultivars to inking appearance, whereas cv. ‘229’ showed resistance to the development of inking symptoms. This finding indicates that, in addition to mechanical damage, genetic background plays a crucial role in determining whether fruits will develop the disorder.

Similar observations have been reported in different peach and nectarine cultivars. In these cases, a close relationship between susceptibility to visual or cosmetic defects and concentration of phenolic compounds has been described, with more susceptible cultivars generally exhibiting higher phenolic contents [4]. These suggest that the differential susceptibility of yellow peach cultivars to showing inking symptoms in the presence of mechanical damage could be related to their phenolic compound contents.

To verify that phenolic compounds are responsible for inking appearance in fruits subjected to mechanical damage, the content of total phenols and chlorogenic acid (principal phenolic compound in peach) was evaluated. Comparison between the three cultivars evaluated showed that at 0 min of brushing, the fruits of cv. ‘MG8’ presented the highest total phenolic content; at 1 min of brushing, the fruits of cv. ‘229’ showed the highest concentration of phenols, and samples of cv. ‘Colegio’ treated for 3 and 5 min exhibited the highest phenol content (Figure 3B). With respect to chlorogenic acid, the three cultivars did not show differences with brushing times of 0 and 1 min; however, when peaches were brushed for 3 and 5 min, the fruits of cv. ‘Colegio’ presented the highest content of chlorogenic acid, followed by ‘MG8’ and ‘229’ (Figure 3C). These results revealed that the peaches of cv. ‘Colegio’, the cultivar more susceptible to inking, showed the highest phenolic compounds content following mechanical damage; in contrast, peaches of the cv. ‘229’ cultivar, with no incidence of inking disorder, consistently showed lower contents of total phenolic and chlorogenic acid in response to brushing. This suggests that phenolic compounds play a key role in inking development; therefore, the low concentration of these compounds in cv. ‘229’ could be the cause of the inking resistance observed, unlike ‘MG8’ and ‘Colegio’ cultivars, which showed a high content of phenolic compounds and elevated susceptibility to inking development.

Importantly, in the three peach cultivars analyzed, fruit brushed for 5 min showed increased total phenols and chlorogenic acid contents in comparison with non-brushed peaches. These results suggest that brushing provoked mechanical damage that activated the phenolic pathway synthesis as a response to repair the damaged tissue. It has been widely reported that phenolic compounds accumulate in fruits as a defense mechanism under stress conditions, including mechanical injury [13].

**Figure 4:**
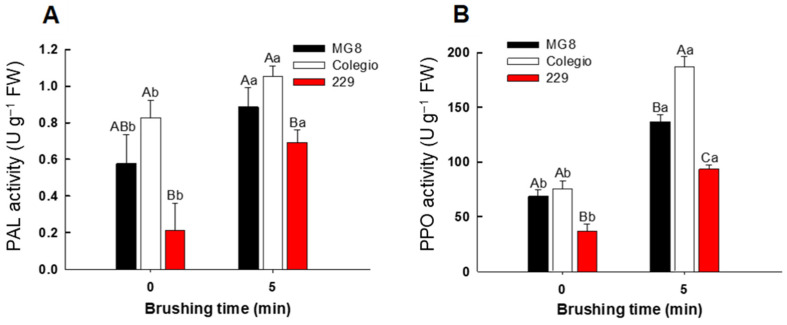
Phenylalanine ammonia-lyase (**A**) and polyphenol oxidase (**B**) activities in epidermis of peach fruit from cultivars ‘MG8’, ‘Colegio’, and ‘229’ subjected to brushing for 0 and 5 min. Graphs depict mean values ± SE (*n* = 3). Letters indicate statistical comparison in the three cultivars using one-way ANOVA and Tukey test (*p <* 0.05). Different capital letters indicate statistical differences between cultivars, and lowercase letters indicate statistical differences within the same cultivar.

As expected, enzymatic activity associated with phenolic biosynthesis showed changes between cultivar and brushing treatment times. Phenylalanine ammonia-lyase (PAL) activity increased differentially into the three cultivars after brushing treatment. The initial activity (0 min of brushing time) in fruit of cv. ‘Colegio’ and ‘MG8’ was higher than the activity in peach cv. ‘229’ (Figure 4A); after 5 min of brushing, PAL activity increased in all cultivars, but fruit of cv. ‘229’ maintained lower values compared with the peaches of cv. ‘Colegio’ and ‘MG8’. These results explain the different content of phenolic compounds observed in the three cultivars, showing that the phenolic compounds content is directly related to the activity of PAL enzyme. The induction of PAL activity under mechanical stress has been reported as an adaptive plant response, promoting the synthesis of phenolic compounds involved in wound healing [12,13,14].

In addition to the increase in the biosynthesis of phenolic compounds, mechanical damage can facilitate contact between phenolic substrates and oxidative enzymes such as polyphenol oxidase (PPO), leading to browning reactions associated with inking disorder. The initial PPO activity (0 min of brushing time) was higher in fruit of cv. ‘Colegio’ and ‘MG8’ compared with peaches of cv. ‘229’. After 5 min of treatment, PPO activity increased significantly in the three cultivars, with the ‘Colegio’ cultivar showing the highest PPO activity compared with ‘MG8’ and ‘229’ (Figure 4B). This result could explain the browning symptoms observed in these fruits after brushing treatment and is associated with the release of phenolic compounds and their subsequent enzymatic oxidation by PPO [12,14,15,16,17]. Interestingly, fruits of cv. ‘229’ exhibited the lowest PPO activity after 5 min of brushing, and inking on epidermis was not observed. This suggests that phenolic accumulation alone is not sufficient to trigger inking development and highlights the importance of phenolic oxidative metabolism. PPO catalyzes the oxidation of phenolic substrates into quinones that subsequently polymerize into dark pigments; therefore, limited PPO activity would restrict browning reactions even in the presence of sufficient substrate [17].

Additionally, differences in cellular compartmentalization may contribute to the observed resistance. In intact tissues, phenolic compounds are typically stored in the vacuole, whereas oxidative enzymes such as PPO are localized in plastids. The development of browning requires a disruption of these compartments, allowing for the interaction of the enzyme and substrate [14]. It is possible that cv. ‘229’ maintains greater cellular integrity under mechanical stress or exhibits structural characteristics that limit the interaction between phenolics and oxidative enzymes, thereby reducing pigment formation.

Taken together, these findings suggest that resistance to inking in cv. ‘229’ is not exclusively determined by phenolic concentration, but could be associated with reduced oxidative enzyme activity and preservation of cellular compartmentalization.

### 2.3. Interaction Between Brushing and Fe(III) Ions on Inking Development

**Figure 5:**
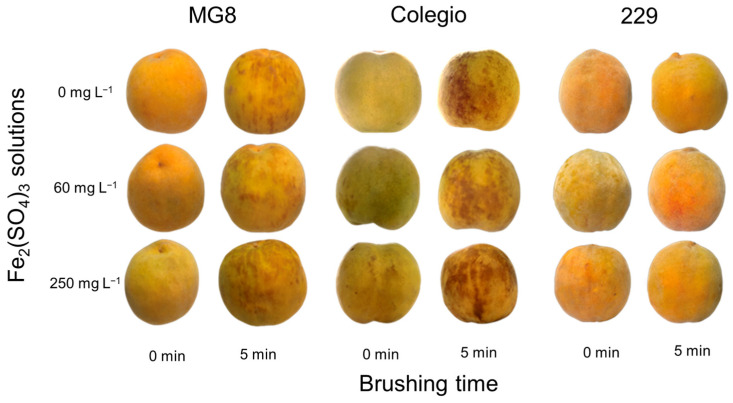
Inking development in three yellow peach cultivars (‘MG8’, Colegio’, and ‘229’) subjected to brushing for 0 and 5 min and spraying with 0, 60, and 250 mg L^−1^ of Fe_2_(SO_4_)_3_ solutions after their storage for 48 h at 1 °C.

Based on preceding results, analyses of the interaction between Fe(III) ions and brushing could provide more information to explain the inking mechanism and the different susceptibility observed in yellow peach cultivars. Peach fruit samples from three cultivars were brushed for 0 and 5 min and sprayed with different concentrations of Fe_2_(SO_4_)_3_ solutions. The three cultivars brushed for 0 min and sprayed with Fe(III) solutions did not show inking development (Figure 5 and Table S4), whereas all fruits from ‘Colegio’ and ‘MG8’ cultivars brushed for 5 min and sprayed with 0, 60, and 250 mg L^−1^ of Fe(III) solutions showed inking development. Importantly, inking development was more evident at a higher concentration of Fe_2_(SO_4_)_3_ solution. This indicates that mechanical damage without Fe presence can induce inking disorder, but the presence of Fe(III) exacerbated the phenomenon’s development. Interestingly, in all treatments, samples from cv. ‘229’ did not show symptoms of this postharvest defect. These results showed that the presence of Fe(III) ions, in addition to mechanical damage by brushing, induced inking development, and fruit response was dependent on the cultivar, mechanical damage applied, and concentration of Fe(III) ions.

The epidermis of the fruit that was not brushed and not sprayed with Fe(III) solutions showed the lowest contents of total phenols and chlorogenic acid (Table 1). On the other hand, non-brushed fruit sprayed with Fe_2_(SO_4_)_3_ (60 and 250 mg L^−1^) showed significantly increased contents of total phenolic compounds and chlorogenic acid in the three cultivars, confirming that the application of Fe(III) to peach fruits induced the biosynthesis of phenolic compounds. This enhanced production may represent a physiological response to scavenging Fe(III) ions, as previously described elsewhere [18]. Additionally, Zhang et al. [19] reported that chlorogenic acid could act as a signaling molecule capable of inducing its own biosynthesis, thereby contributing to wound healing responses in mechanically damaged tissues.

These results show that spraying peach fruits with 60 or 250 mg L^−1^ of Fe_2_(SO_4_)_3_ and brushing for 5 min induced an increase in phenolic compounds and chlorogenic acid, causing a more intense inking development in peaches of cv. ‘Colegio’ and cv. ‘MG8’. Interestingly, fruits of cv. ‘229’ did not show an inking defect, although peaches of this cultivar showed a similar synthesis of chlorogenic acid with the Fe_2_(SO_4_)_3_ treatments. This indicates that an increase in phenols does not generate an inking appearance, but the enzymatic activity that provokes oxidation of these compounds could cause the phenomenon’s symptoms.

Our results are consistent with observations reported by Cheng and Crisosto [8], who studied inking development in pigmented free-stone peach cultivars (‘My Glo’, ‘Flavorcrest’, ‘O Henry’, and ‘Elegant Lady’ cultivars) subjected to Fe treatments combined with mechanical damage. In red cultivars, anthocyanins were identified as the primary compounds forming dark iron–pigment complexes responsible for inking. In contrast, the yellow peach cultivars evaluated in the present study do not synthesize anthocyanins; therefore, the formation of Fe–chlorogenic acid or Fe–phenolic complexes could be associated with inking development in these fruits.

In this context, Fe(III) ions could act as a superficial contaminant that induces phenolic biosynthesis as part of a stress response, resulting in the formation of complexes that are subsequently oxidized, generating the characteristic black spots associated with inking. It is important to note that micronutrients such as Fe, Zn, Mn, Cu, B, Ni, and Mo are frequently supplied to plants through foliar fertilization. During foliar applications, these elements may be deposited on the fruit surface, potentially inducing phenolic biosynthesis. These phenolic compounds can react superficially with metal ions to form Fe–phenolic complexes [20], which may be oxidized by PPO after mechanical damage, thus triggering inking development. Although some chemical elements, such as aluminum or copper, may also act as contaminants, certain ions can be absorbed or metabolized without inducing external defects [21].

Previous studies demonstrated a rapid formation of Fe–chlorogenic acid complexes as the ferric sulfate concentration increased from 0 to 0.7 mM [8], which falls within the concentration range evaluated in the present study. These reactions may represent part of the normal physiological mechanisms involved in iron acquisition and transport in plants. Peach trees grown in calcareous soils (pH ≈ 7.9) frequently suffer from iron deficiency or iron chlorosis due to low Fe bioavailability [22].

Iron plays a crucial role in photosynthesis and other metabolic processes, and its uptake and homeostasis are tightly regulated in plants [23]. Under conditions of limited Fe availability, plants activate mechanisms to enhance Fe acquisition and transport, including the formation of Fe–phenol or Fe–chlorogenic acid complexes that facilitate the reduction of Fe^3+^ to the more bioavailable Fe^2+^ form [20,24].

Based on these observations, our data indicate that inking development in peach fruits may be linked to physiological mechanisms involved in iron acquisition, specifically the formation of phenolic–Fe complexes. Following mechanical damage, these complexes are enzymatically oxidized, resulting in the formation of dark spots in fruit skin.

This study confirms that both mechanical damage and the presence of Fe(III) ions are key factors in inking development, in agreement with previous reports [6,7,8]. However, our findings suggest that inking may represent a secondary consequence of normal iron uptake mediated by phenolic compounds.

Finally, clear varietal differences were observed. Fruit from cv. ‘229’ did not develop surface darkening under any treatment, likely due to its lower phenolic accumulation and reduced PPO activity following mechanical damage. It should be noted that differences in epidermis thickness and firmness among cultivars may influence the degree of mechanical damage induced under identical brushing conditions. However, all fruits were subjected to the same standardized treatment, allowing for a controlled comparison of cultivar responses. Therefore, the observed differences in inking development are more likely associated with cultivar-dependent physiological and biochemical responses rather than the extent of physical damage. Further studies focusing specifically on structural traits such as epidermis thickness and firmness would be valuable to better understand their contribution to inking susceptibility.

## 3. Materials and Methods

### 3.1. Peach Fruit Origin

Clingstone yellow peach fruit (*Prunus persica* L.) cv. ‘MG8’, cv. ‘Colegio’, and cv. ‘229’ were collected from a commercial orchard located at Aguascalientes, Mexico (22°05′26″ N, 102°05′58″ W), in June, July and August 2021, respectively. Fruits at commercial maturity, based on ground color, were transported in a cooler filled with ice packs to the laboratory located in Queretaro, Mexico (4 h to arrive). Immediately after arrival, fruits were stored at 1 °C for 24 h.

### 3.2. Mineral Analysis

To this analysis, for each cultivar, fruit with inking and non-inking were selected, except for cv. ‘229’, because only non-inking fruits were available from this cultivar. The epidermis of the three varieties was carefully removed using a surgical bistoury and then it was lyophilized. Mineral analysis was carried out according to the methodology proposed by Altarawneh [25]. One gram of lyophilized sample was treated with 10 mL of an acid mixture (67% HNO_3_, 65% HClO_4_; 2:1) and heated for 45 min at 90 °C; then, it was heated at 150 °C for 10 °C/min rate, and finally, the reaction mixture was left at 150 °C for two hours. After the mixture was cooled and filtered; the filtrate was diluted with deionized water and analyzed in a spectrophotometer of atomic absorption (AAnalyst 300, PerkinElmer, Waltham, MA, USA). The content of Fe, Zn, Mn, Cu, B, Ni, and Mo was estimated using a standard curve for each mineral.

### 3.3. Relationship Between Fe Presence and Inking Development in Yellow Peach Cultivars

Peach fruits (*n* = 48) without an inking defect were washed with distilled water and placed on a flat surface to dry. Then, fruits were sprayed with different concentrations of Fe_2_(SO_4_)_3_ (0, 60, and 250 mg L^−1^); the pH of Fe_2_(SO_4_)_3_ solutions ranged between 2.3 and 2.6; after this treatment, peach fruits were stored for 48 h at 1 °C, and visual appearance and total phenolic content were assessed.

### 3.4. Relationship Between Mechanical Damage and Inking Disorder in Yellow Peach Cultivars

Peach fruits (*n* = 32) without visible inking symptoms were subjected to controlled mechanical damage using a custom-built brushing device designed to simulate, at laboratory scale, commercial brushing systems used during postharvest handling. The system consisted of five sequential rotating rollers equipped with soft nylon (polyamide) bristles, like those used in commercial peach packing lines. The rollers operated at 40 rpm and were connected through a gear mechanism, ensuring continuous and uniform contact between the fruit surface and the brushing elements. Fruits were placed on the rollers and allowed to move freely along the system, ensuring homogeneous exposure to brushing. Each treatment was performed using independent biological replicates. After brushing, fruits were stored at 1 °C for 48 h prior to analysis. Subsequently, phenolic compounds and enzymatic activities were determined in the fruit epidermis.

### 3.5. Effect of Fe Ions and Mechanical Damage Interaction in Inking Appearance

Fruits (*n* = 48) of each cultivar were divided into groups according to different brushing times (0 and 5 min) and Fe_2_(SO_4_)_3_ solutions (0, 60 and 250 mg L^−1^). Non-treated fruits (0 min of brushing time and sprayed with 0 mg L^−1^ of Fe_2_(SO_4_)_3_ solution) were taken as the control group. Each brushed fruit group was sprayed with the Fe_2_(SO_4_)_3_ solutions. Fruits were stored for 48 h at 1 °C. Total phenolic compounds and chlorogenic acid were measured in the epidermis of these fruits after 0 and 48 h.

### 3.6. Visual Analysis and Color Measurement

Inking was assessed by visual inspection based on the presence or absence of dark discoloration in the fruit epidermis. Fruits showing visible darkening were classified as “inking”, whereas fruits without symptoms were classified as “non-inking”.

In addition, color measurements (L* parameter) were recorded with a colorimeter CM-600D (Konica Minolta, Tokyo, Japan) to support the visual classification, and these data are presented in the Supplementary Materials.

### 3.7. Phenolic Compounds Extraction

To evaluate phenolic compounds, a methanolic extract was prepared. To 2 g of epidermis samples, 25 mL of methanol 80% (*v*/*v*) was added, and this mixture was homogenized with an Ultraturrax (T-25, IKA, Staufen, Germany) at 10,000 rpm for 1 min; the homogenized extract was centrifuged at 12,000 rpm during 15 min at 4 °C; then, the supernatant obtained was filtered (Whatman #4) and subjected to spectrophotometric analyses and HPLC separation; three independent extracts were prepared, and each extract was analyzed in triplicate.

### 3.8. Quantification of Total Phenol Content

Total phenolic content was assessed according to the Folin–Ciocalteu method [26]. To 400 µL of phenolic extract, 1 mL of distilled water and 200 µL of Folin–Ciocalteu reagent were added; this mixture settled for 5 min. Then, 2 mL of 7% of sodium carbonate solution and 1.4 mL of distilled water were aggregated with it to obtain the final mixture reaction. It was kept at room temperature and protected from light for 1 h. Finally, absorbance was measured using a spectrophotometer (Lambda 365, PerkinElmer, Waltham, MA, USA) at 750 nm. A gallic acid standard curve was used for the quantification of total phenolics, and the results were reported as equivalents of gallic acid (GAE) per 100 g of sample fresh.

### 3.9. Phenolic Profile Analysis

Chlorogenic acid was analyzed with the HPLC method. It used equipment (Waters, Milford, MA, USA) comprising an Alliance e2695 quaternary pump and a 2998 PDA detector, controlled by the Empower3 software (Feature Release 1, Waters, Milford, MA, USA). Thirty microliters of methanolic extract were injected into the pump to separate phenolic compounds using a symmetry C-18 (5 μm, 150 mm × 4.6 mm) column at 35 °C. The mobile phase consisted of solvent A [water/formic acid (99.9:0.1, *v*/*v*)] and solvent B (acetonitrile), with a flow rate of 0.5 mL min^−1^. The gradient used was as follows: 0 min, 2% B; 0–40 min, 20% B; 40–45 min, 100% B; and 45–60 min, 2% B. Chlorogenic acid detection was performed at 320 nm, and identification was carried out by retention time comparison and UV–VIS data comparison with standard (Sigma-Aldrich, St. Louis, MO, USA). A chlorogenic acid standard calibration curve was used for quantitation.

### 3.10. Phenylalanine Ammonium Lyase (PAL) Activity

To evaluate PAL activity, first an enzymatic extract was prepared. Epidermis samples (4 g) were homogenized in an Ultraturrax (T-25, IKA, Staufen, Germany) for 2 min with 16 mL of 50 mM borate buffer (pH 8.5), 14 µL of 0.01 M β-mercaptoethanol, and 0.4 g of PVPP. Then, the homogenate was centrifuged for 20 min at 12,000 rpm at 4 °C. To estimate the enzyme activity, a reaction was performed according to a method reported elsewhere [27]. The reaction mixture consisted of 2.7 mL of enzymatic extract and 300 µL of 100 mM phenylalanine. After 5 min and 60 min of incubation at 40 °C, absorbance was measured at 290 nm (Lambda 365, PerkinElmer, Waltham, MA, USA). Enzyme activity was estimated using a standard curve of cinnamic acid. One unit of enzymatic activity was defined as 1 μmol of cinnamic acid produced in one hour. Enzymatic activity was reported as U of PAL g^−1^ of fresh tissue; three independent extracts were prepared, and each extract was analyzed in triplicate.

### 3.11. Polyphenol Oxidase (PPO) Activity

For enzyme extraction, peach epidermis samples (5 g) were homogenized in an Ultraturrax (T-25, IKA, Staufen, Germany) for 2 min with 20 mL, 0.05 M phosphate buffer (pH 6.0), and 0.6 g of PVPP. The mixture was centrifuged for 15 min at 10,000 rpm at 4 °C. The activity of PPO was evaluated according to the methodology of Fang et al. [28], with modifications. The reaction mixture consisted of 100 µL of enzyme extract and 50 µL of 60 mM catechol as a phenolic substrate. Absorbance was measured over 3 min at 420 nm (Lambda 365, PerkinElmer, Waltham, MA, USA). One unit of PPO (U) was defined as the amount of enzyme necessary to generate an absorption increase of 0.001 units for each minute of reaction time. Enzymatic activity was estimated using the equation below (Equation (1)) and was reported as U of PPO g^−1^ of fresh tissue; three independent extracts were prepared, and each extract was analyzed in triplicate.

U of PPO per g of fresh tissue was estimated considering the grams of sample and milliliters of buffer used.

(1) $$Activity\;\left({U.ml}^{-1}\right)=\left[\left({AF}_{sample}-{AI}_{sample}\right)-\left({AF}_{blank}-{AI}_{blank}\right)\right]/(0.001\;\times \;t)$$

where *AF_sample_* corresponds to sample absorbance final; *AI_sample_* to sample absorbance initial; *AF_blank_* to blank absorbance final; *AI_blank_* to blank absorbance initial; and *t* to time (min).

### 3.12. Statistical Analysis

A completely randomized unifactorial design was used. Each treatment consisted of 12 fruits (*n* = 12). For phenolic and enzymatic analyses, three independent extracts were prepared from each fruit sample, and each extract was analyzed in triplicate. Comparisons between inked and non-inked fruits were performed using Student’s *t*-test. One-way analysis of variance (ANOVA) followed by Tukey’s test was used to evaluate the effects of brushing time and Fe_2_(SO_4_)_3_ concentration on inking development and related biochemical parameters. In addition, a two-way ANOVA was performed to assess the interaction between mechanical damage (brushing time) and Fe_2_(SO_4_)_3_ concentration.

Prior to statistical analysis, data were tested for normality and homogeneity of variance. Statistical analyses were conducted using JMP 6.0 software (SAS Institute Inc., Cary, NC, USA), and significance was established at *p* < 0.05.

## 4. Conclusions

This research revealed that application of Fe(III) sulfate solutions induced the biosynthesis of phenolic compounds; however, it does not induce inking appearance; therefore, Fe presence in peach epidermis is not the direct cause of inking disorder. Likewise, mechanical damage also induced the biosynthesis of phenolic compounds, as well PAL and PPO activities, generating the inking disorder in ‘Colegio’ and ‘MG8’ cultivars, and this defect was exacerbated by the Fe(III) ions’ presence. Notably, peach fruit from cv. ‘229’ did not develop inking, indicating resistance to this disorder. The disorder is closely linked to cultivar-dependent differences in phenolic metabolism and oxidative enzyme activity. The resistance observed in ‘229’ highlights the importance of the intrinsic metabolic traits of each cultivar in determining susceptibility or resistance to inking disorder. Taken together, it is shown that inking development follows a sequential process in which Fe(III) ions first stimulate phenolic accumulation, then mechanical damage subsequently disrupts cellular compartmentalization, and finally enzymatic oxidation of phenolic compounds leads to the formation of dark pigments. These findings provide a physiological basis for improving postharvest handling practices and for selecting cultivars with reduced susceptibility to this disorder.

## Figures and Tables

**Table 1:** Total phenolic and chlorogenic acid contents in clingstone peach epidermis from cultivars ‘MG8’, ‘Colegio’, and ‘229’ subjected to brushing and superficial spraying with Fe_2_(SO_4_)_3_ solutions.

Cultivar	Brushing Time (min)	Fe_2_(SO_4_)_3_ (mg L^−1^)	Total Phenolic (mg eq. of Gallic Acid/100 g FW)	Chlorogenic Acid (mg CA/100 g FW)
MG8	0	0	99.0 ± 4.0 ^d^	9.36 ± 1.4 ^c^
		60	137.8 ± 2.3 ^b^	16.2 ± 3.4 ^abc^
		250	156.1 ± 2.3 ^a^	21.1 ± 0.4 ^a^
	5	0	111.1 ± 7.2 ^c^	16.9 ± 1.6 ^ab^
		60	138.1 ± 12.1 ^b^	11.8 ± 4.5 ^bc^
		250	159.3 ± 11.1 ^a^	23.4 ± 2.8 ^a^
Colegio	0	0	69.5 ± 8.3 ^c^	7.14 ± 0.9 ^c^
		60	130.5 ± 19.6 ^b^	16.41 ± 2.7 ^ab^
		250	145.9 ± 25.5 ^ab^	19.47 ± 3.1 ^ab^
	5	0	151.1 ± 10.4 ^ab^	19.31 ± 3.4 ^ab^
		60	155.5 ± 9.0 ^ab^	12.66 ± 3.8 ^bc^
		250	161.2 ± 28.6 ^a^	22.52 ± 3.2 ^a^
229	0	0	71.8 ± 6.7 ^c^	8.09 ± 0.8 ^c^
		60	131.8 ± 5.7 ^a^	15.05 ± 2.5 ^ab^
		250	134.8 ± 8.5 ^a^	19.22 ± 3.5 ^a^
	5	0	111.1 ± 7.2 ^b^	11.38 ± 1.5 ^bc^
		60	126.4 ± 8.7 ^ab^	11.99 ± 1.1 ^bc^
		250	130.5 ± 24.1 ^a^	12.02 ± 3.2 ^bc^

Total phenolic content: data are expressed as gallic acid equivalents. CA: chlorogenic acid. Mean of three replicates ± SE. Letters indicate statistical comparison in the three cultivars using two-way ANOVA and Tukey test (*p <* 0.05). Different lowercase letters indicate statistical differences within the same cultivar.

## Data Availability

The original contributions presented in this study are included in the article/Supplementary Material. Further inquiries can be directed to the corresponding author.

## Supplementary Materials

The following supporting information can be downloaded at: https://www.mdpi.com/article/10.3390/ijms27083681/s1.
