# Supplementary material for: Susceptibility of Yellow Clingstone Peach Cultivars to Inking Disorder
Source: Int J Mol Sci. 2026 Apr 21;27(8):3681. doi: 10.3390/ijms27083681 (PMC13116263; doi:10.3390/ijms27083681)
Supplement: Supplementary file 1 [file ijms-27-03681-s001.zip › ijms-4186925-supplementary.pdf]

Table of Contents

Supplemental tables

Table S1. Table S1. Mineral content of peach fruit epidermis in inked and non-inked samples.

Table S2. Lightness (L*) values of peach fruit epidermis subjected to different $Fe_2(SO_4)_3$ concentrations.

Table S3. Lightness (L*) values of peach fruit epidermis subjected to different brushing times.

Table S4. Lightness (L*) values of peach fruit epidermis subjected to different brushing times and superficial spraying with $Fe_2(SO_4)_3$ solutions.

Table S1. Mineral content of peach fruit epidermis in inked and non-inked samples.

**Mineral content**	**MG8**		**Colegio**		**229**
mg Kg^{-1}	Inked	Non-inked	Inked	Non-inked	Non-inked
Zinc (Zn)	21.7 ± 3.8	18.4 ± 2.7	34.3 ± 1.6*	19.7 ± 1.5	21.8 ± 2.5
Manganese (Mn)	38.9 ± 1.8	37.3 ± 2.2	38.8 ± 3.7	36.8 ± 3.4	39.0 ± 3.0
Copper (Cu)	3.36 ± 1.7*	7.12 ± 1.9	3.16 ± 0.9	5.29 ± 1.2	2.62 ± 0.7
Boron (B)	45.9 ± 4.4*	36.7 ± 3.6	45.9 ± 2.9*	39.4 ± 2.0	47.2 ± 5.2
Nickel (Ni)	0.03 ± 0.007*	0.08 ± 0.005	0.19 ± 0.05	0.1 ± 0.04	0.07 ± 0.008
Molybdenum (Mo)	0.09 ± 0.01	0.08 ± 0.006	0.09 ± 0.009	0.08 ± 0.02	0.13 ± 0.01

Values are expressed as mean ± standard deviation (n = 3). Comparisons between inked and non-inked fruits within each cultivar were performed using Student's *t*-test. Asterisks (*) indicate significant differences between inking and non-inking samples (P < 0.05).

Table S2. Lightness (L*) values of peach fruit epidermis subjected to different $Fe_2(SO_4)_3$ concentrations.

	**$Fe_2(SO_4)_3$ concentrations**		
**Cultivars**	0 mg L^{-1}	60 mg L^{-1}	250 mg L^{-1}
MG8	58.43 ± 0.8^{a}	58.69 ± 0.8^{a}	58.61 ± 0.8^{a}
Colegio	61.11 ± 0.6^{a}	61.70 ± 0.6^{a}	61.92 ± 0.6^{a}
299	59.37 ± 0.7^{a}	57.77 ± 0.7^{a}	58.44 ± 0.7^{a}

Values are expressed as mean ± standard deviation (n = 12). Different letters indicate significant differences (P < 0.05).

Table S3. Lightness (L*) values of peach fruit epidermis subjected to different brushing times.

	**Brushing time**			
**Cultivars**	0 min	1 min	3 min	5 min
MG8	60.4 ± 0.7^{a}	58.3 ± 0.7^{a}	59.1 ± 0.7^{a}	56.6 ± 0.7^{b}
Colegio	63.9 ± 0.8^{a}	62.5 ± 0.8^{ab}	59.1 ± 0.8^{c}	59.9 ± 0.8^{bc}
299	60.9 ± 0.7^{a}	58.6 ± 0.7^{a}	58.6 ± 0.7^{a}	60.3 ± 0.7^{a}

Values are expressed as mean ± standard deviation (n = 12). Different letters indicate significant differences between brushing times within the same cultivar (P < 0.05).

Table S4. Lightness (L*) values of peach fruit epidermis subjected to different brushing times and superficial spraying with $Fe_2(SO_4)_3$ solutions.

Cultivar	Brushing time (min)	$Fe_2(SO_4)_3$ (mg L^{-1})	L*
MG8	0	0	66.2 ± 0.9[a]
		60	64.6 ± 0.9[a]
		250	64.0 ± 0.9[ab]
	5	0	60.4 ± 0.9[b]
		60	56.1 ± 0.9[c]
		250	56.0 ± 0.9[c]
Colegio	0	0	60.7 ± 1.0[a]
		60	60.6 ± 1.0[a]
		250	61.2 ± 1.0[a]
	5	0	51.3 ± 1.0[c]
		60	57.9 ± 1.0[b]
		250	50.0 ± 1.0[c]
229	0	0	60.8 ± 0.6[ab]
		60	60.6 ± 0.6[ab]
		250	61.2 ± 0.6[ab]
	5	0	61.7 ± 0.6[a]
		60	59.4 ± 0.6[ab]
		250	58.9 ± 0.6[b]

Values are expressed as mean ± standard deviation (n = 12). Data were analyzed separately for each cultivar using two-way ANOVA, considering brushing time and $Fe_2(SO_4)_3$ concentration as factors. Different letters indicate significant differences among treatments within each cultivar ($P < 0.05$), according to Tukey's test.
